# Edema associated with Haloperidol: A Rare Observation

**DOI:** 10.1192/j.eurpsy.2025.2081

**Published:** 2025-08-26

**Authors:** R. Jbir, F. Guermazi, N. Bouattour, D. Mnif, R. Masmoudi, I. Bouaziz, R. Atheymen, K. Ksouda, K. Zghal, I. Baati, J. Masmoudi

**Affiliations:** 1 Psychiatry ‘A’ deprtment, Hedi Chaker university hospital; 2 Regional Pharmacovigilance Center, faculty of medicine of sfax, Sfax, Tunisia

## Abstract

**Introduction:**

Haloperidol (Haldol®) is first-generation antipsychotic that still have a place in the treatment of schizophrenia. However, this molecule is associated with numerous side effects, particularly extrapyramidal symptoms. Edema has been rarely described with haloperidol, and may have an immunoallergic or pharmacodynamic mechanism.

**Objectives:**

Our aim is to study a rare case of facial and limb edema attributed to haloperidol.

**Methods:**

We report the case of a 22-year-old female patient with schizophrenia who developed facial and limb edema after taking haloperidol.

**Results:**

We report the case of a 22-year-old woman. She was admitted to our psychiatric department “A” as an involuntary inpatient after she threatened to stab her father. She was diagnosed with schizophrenia. The patient has no past medical history. The somatic examination and the admission report were correct.

Given the need for a slow-release neuroleptic, the patient was treated with haloperidol in gradually increasing doses up to 30 mg per day.

Six weeks after starting treatment, the patient developed progressive edema of the face, eyelids and all 4 limbs, with a marked increase in weight and body mass index from 31 to 36.6.

The patient was examined by internists. An etiologic investigation of this edema was initiated, excluding renal origin (strictly normal renal work-up and negative proteinuria), cardiac origin (in view of a negative pro-brain natriuretic peptide, D-dimer and troponins and a normal cardiac echography), hepatic (in the presence of a strictly normal work-up including: protidemia, albuminemia, transaminases, PAL, GGT, bilirubin and prothrombin levels) and endocrine (a normal thyroid work-up) origin.

The edema progressively worsened on haloperidol over a period of 10 days. A drug-induced origin was suspected. We contacted the regional pharmacovigilance center in Sfax, which incriminated haloperidol and recommended its immediate discontinuation.

After discontinuing the drug, the edema regressed progressively until it disappeared completely after 15 days, confirming that haloperidol was the drug responsible and contraindicating its further use.

The responsibility of haloperidol in the genesis of the edema was retained with a plausible score (C2S2I2B3). The scores were calculated according to the French Bégaud method.

**Conclusions:**

Edema is not a common side effect of typical antipsychotics, especially haloperidol. However, it is important to emphasize that this effect, although rare, can occur. Clinicians are advised to be aware of edema in all patients taking first or second antipsychotics.

**Disclosure of Interest:**

None Declared

